# Lower Respiratory Tract Infections Following Respiratory Syncytial Virus Monoclonal Antibody Nirsevimab Immunization Versus Placebo: Analysis From a Phase 3 Randomized Clinical Trial (MELODY)

**DOI:** 10.1093/cid/ciae596

**Published:** 2024-12-04

**Authors:** Doug Arbetter, Vancheswaran Gopalakrishnan, Anastasia A Aksyuk, Bahar Ahani, Yue Chang, Ron Dagan, Mark T Esser, Laura L Hammitt, Vaishali S Mankad, Xavier Saez-Llorens, David Shen, Amanda Leach, Elizabeth J Kelly, Tonya Villafana, Deidre Wilkins

**Affiliations:** Biometrics, Vaccines & Immune Therapies, BioPharmaceuticals R&D, AstraZeneca, Boston, Massachusetts, USA; Bioinformatics, Vaccines & Immune Therapies, BioPharmaceuticals R&D, AstraZeneca, Gaithersburg, Maryland, USA; Translational Medicine, Vaccines & Immune Therapies, BioPharmaceuticals R&D, AstraZeneca, Gaithersburg, Maryland, USA; Bioinformatics, Vaccines & Immune Therapies, BioPharmaceuticals R&D, AstraZeneca, Gaithersburg, Maryland, USA; Biometrics, Vaccines & Immune Therapies, BioPharmaceuticals R&D, AstraZeneca, Gaithersburg, Maryland, USA; Shraga Segal Department of Microbiology, Immunology and Genetics, Faculty of Health Sciences of the Ben-Gurion University of the Negev, Beer-Sheva, Israel; Vaccines & Immune Therapies, BioPharmaceuticals R&D, AstraZeneca, Gaithersburg, Maryland, USA; Department of International Health, Johns Hopkins University, Baltimore, Maryland, USA; Clinical Development, Respiratory & Immunology, BioPharmaceuticals R&D, AstraZeneca, Durham, North Carolina, USA; Cevaxin Research Center, Panama City, Panama; Department of Infectious Diseases, Hospital del Niño Dr José Renán Esquivel, Panama City, Panama; SNI, National Secretariat of Science, Technology and Innovation, Panama City, Panama; Biometrics, Vaccines & Immune Therapies, BioPharmaceuticals R&D, AstraZeneca, Boston, Massachusetts, USA; Clinical Development, Vaccines & Immune Therapies, BioPharmaceuticals R&D, AstraZeneca, Gaithersburg, Maryland, USA; Translational Medicine, Vaccines & Immune Therapies, BioPharmaceuticals R&D, AstraZeneca, Gaithersburg, Maryland, USA; Vaccines & Immune Therapies, BioPharmaceuticals R&D, AstraZeneca, Gaithersburg, Maryland, USA; Translational Medicine, Vaccines & Immune Therapies, BioPharmaceuticals R&D, AstraZeneca, Gaithersburg, Maryland, USA

**Keywords:** lower respiratory tract infection, nirsevimab, RSV immunization, respiratory viruses

## Abstract

**Background:**

Nirsevimab is an extended half-life, highly potent, anti-respiratory syncytial virus (RSV) fusion protein neutralizing monoclonal antibody with efficacy against RSV-associated medically attended (MA) lower respiratory tract infection (LRTI) in infants and medically vulnerable children (aged ≤24 months). This post hoc exploratory analysis examined the incidence of LRTI from RSV and other respiratory pathogens during MELODY: a 2:1 randomized, double-blind, placebo-controlled, phase 3 study of nirsevimab in healthy term and late preterm (ie, gestational age ≥35 weeks) infants entering their first RSV season.

**Methods:**

A total of 3012 participants were randomized to nirsevimab (n = 2009) or placebo (n = 1003). Nasopharyngeal swabs were collected from infants who presented with an LRTI and tested for 22 different respiratory pathogens using the BioFire^®^ Respiratory 2.1 Panel. Incidence of RSV and non-RSV MA-LRTIs through day 511 and LRTI severity were assessed.

**Results:**

A total of 852 nasopharyngeal swabs were collected from 561 participants through day 511: 519 swabs from 337 nirsevimab participants and 333 swabs from 224 placebo participants. RSV and non-RSV infections were detected in 193 of 852 (22.7%) and 55 of 852 (64.7%) swabs, respectively. RSV infection rates were lower with nirsevimab compared with placebo, including RSV–rhinovirus/enterovirus coinfections. Rates of other viral infections were similar between study arms. Approximately 70% of single RSV infections and RSV coinfections were adjudicated as mild, and 26.2% of single RSV infections and 24.5% of RSV coinfections required hospitalization.

**Conclusions:**

Nirsevimab protected against RSV single and coinfections, with no evidence of replacement of RSV with other respiratory viruses.

**Clinical Trials Registration.** NCT03979313.

## Teaser

This analysis of lower respiratory tract infections showed that nirsevimab prophylaxis led to lower incidences of respiratory syncytial virus infections over time versus placebo without accompanying increases in incidences of other respiratory infections, demonstrating no evidence of viral replacement.

## Graphical Abstract

**Graphical Abstract:**
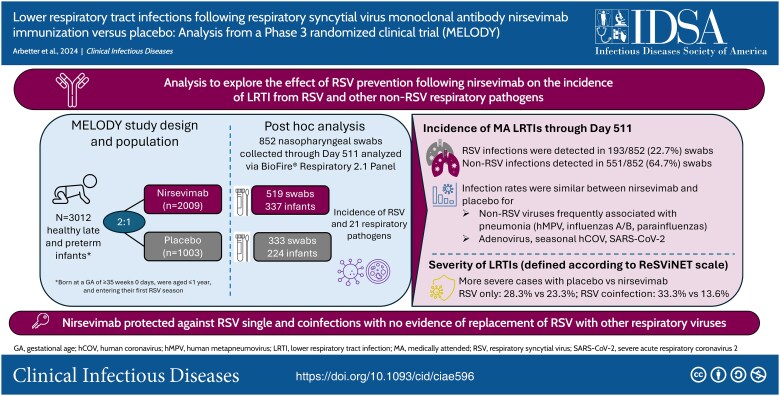
This graphical abstract is also available at Tidbit: https://tidbitapp.io/institutional-portal/clinical-infectious-diseases/tidbits/lower-respiratory-tract-infections-following-respiratory-syncytial-virus-monoclonal-antibody-nirsevimab-immunization-versus-placebo-analysis-from-a-phase-3-randomized-clinical-trial-melody/update

Lower respiratory tract infections (LRTIs) are significant sources of global pediatric morbidity and mortality [1]. Respiratory syncytial virus (RSV) is the leading viral cause of LRTI, with annual RSV epidemics resulting in high levels of healthcare resource utilization among infants and children aged ≤24 months [2, 3]. The risk of severe RSV LRTI is highest during the first year of life, with the majority of hospitalizations occurring in otherwise healthy infants born at term [3, 4].

Nirsevimab is an extended half-life (approximately 71 days) anti-RSV fusion protein monoclonal antibody designed to confer protection against RSV-associated lower respiratory tract disease for the duration of a typical 5-month (150-day) RSV season with a single intramuscular injection [5, 6]. Nirsevimab binds to a conserved antigenic site Ø (“zero”) neutralization epitope exclusive to the prefusion conformation of the RSV fusion protein to inhibit host cell entry [6]. Nirsevimab demonstrated consistently high efficacy against medically attended (MA) RSV LRTI through 150 days post-dose during 2 pivotal placebo-controlled studies. Efficacy rates were 70.1% in healthy preterm infants (gestational age [GA], ≥29 to <35 weeks) during a phase 2b study (NCT02878330) and 76.4% in healthy term and late preterm (GA, ≥35 weeks) infants in an analysis of the full phase 3 MELODY study (NCT03979313) enrollment cohort [7, 8]. A similarly high efficacy against RSV LRTI hospitalization of 83.2% was observed in these populations during the phase 3b HARMONIE study (NCT05437510; open-label trial of nirsevimab versus standard care) [9]. Nirsevimab has been approved for the prevention of RSV lower respiratory tract disease in neonates and infants born during or entering their first RSV season and in children aged ≤24 months who remain vulnerable to severe RSV disease through their second RSV season in various geographic regions [10].

RSV is often present as a coinfection with other respiratory pathogens [11]. Nirsevimab has demonstrated a consistent benefit against MA-LRTI of any cause in placebo-controlled studies during the first RSV season (through 150 days post-dose) and in real-world settings [7, 8, 12, 13]. Questions remain over the contribution of other respiratory pathogens to pediatric LRTIs, especially considering the effects of nirsevimab in reducing RSV-associated lower respiratory disease [14]. In this exploratory post hoc analysis of the MELODY study, we examined the incidence of RSV and non-RSV LRTIs among study participants to explore the effect of RSV prevention on non-RSV LRTI incidence.

## METHODS

### Study Design and Oversight

MELODY was a 2:1 randomized, double-blind, placebo-controlled, phase 3 study to assess the safety and efficacy of nirsevimab in healthy term and late preterm infants. Full details have been published previously [8, 12]. The trial was performed in accordance with the principles of the Declaration of Helsinki and the International Council for Harmonisation Good Clinical Practice guidelines. The protocol was approved by institutional ethics review boards or ethics committees at each trial site. Parents/guardians of participants provided written informed consent.

### Participants

Infants were eligible for inclusion if they were healthy, had been born at a GA of ≥35 weeks 0 days, were aged ≤1 year, and were entering their first RSV season. Key exclusion criteria were history of LRTI/RSV infection or active LRTI/RSV infection prior to or at the time of randomization. Participant baseline characteristics and study inclusion/exclusion criteria have been published previously [8, 12].

Enrollment for the study occurred between 23 July 2019 and 22 October 2021 and was paused between 15 March 2020 and 9 April 2021 due to the coronavirus disease 2019 (COVID-19) pandemic. There were 211 sites in 31 countries (186 sites in 25 countries in the Northern Hemisphere, 25 sites in 6 countries in the Southern Hemisphere) [8, 12]. Participants enrolled prior to the pandemic recruitment pause comprised the primary cohort (n = 1490), while the full enrollment cohort comprised 3012 participants (nirsevimab n = 2009; placebo n = 1003) [8].

### Procedures and Assessments

Participants were randomized to receive a single intramuscular injection of nirsevimab (weight-banded dosing: 50 mg if infant weight <5 kg, 100 mg if ≥5 kg) or saline placebo prior to or during their first RSV season [8, 12]. All participants were under continuous passive surveillance for MA RSV LRTI from day 1 through day 511 (ie, 510 days post-dose), with days 1–151 defined as participant's first RSV season, days 152–361 as the interim period between RSV seasons, and days 362–511 as the participant's second RSV season. Nasopharyngeal swabs were collected from participants who received medical attention for a respiratory illness as part of routine clinical care in inpatient and outpatient settings and within approximately 2 days of initial healthcare provider assessment and diagnosis. RSV infection was confirmed by quantitative real-time reverse-transcription polymerase chain reaction assay (RT-PCR; Lyra^®^ RSV + human metapneumovirus [hMPV] assay, Quidel, San Diego, CA) in a central laboratory (Eurofins-Viracor, Columbia, MO) [8, 12]. Cycle threshold (CT) values are representative of participants’ viral loads. Supplementary Table 1 lists criteria for meeting the primary case definition of MA RSV LRTI.

Participant swabs were also analyzed for non-RSV LRTIs using the BioFire^®^ Respiratory 2.1 Panel (bioMérieux, Salt Lake City, UT), a real-time, nested, multiplexed nucleic acid amplification test intended for the simultaneous qualitative detection and differentiation of nucleic acids from 22 viruses and bacteria associated with respiratory tract infections from a single nasopharyngeal swab [15]. The BioFire Panel detects the following respiratory virus and subtypes: RSV, adenovirus (AdV), human rhinovirus/enterovirus, severe acute respiratory coronavirus 2 (SARS-CoV-2), seasonal human coronaviruses (hCoV; 229E, HKU1, NL63, OC43), hMPV, influenza A/B (influenza A virus, influenza A virus A/H1, influenza A virus A/H3, influenza A virus A/H1–2009, and influenza B virus), parainfluenza viruses 1–4, and the following bacterial pathogens: *Bordetella pertussis*, *Bordetella parapertussis*, *Chlamydophila pneumoniae*, and *Mycoplasma pneumoniae*. The incidence of hMPV, influenza A/B, and parainfluenza LRTIs was assessed together as viruses frequently associated with pediatric pneumonia (VFAPn) [16].

### Outcomes

This post hoc exploratory analysis assessed the incidence of all MA RSV and non-RSV LRTIs through day 511, which were further subcategorized according to LRTI severity, defined according to the Respiratory Syncytial Virus Network (ReSViNET) scale (Supplementary Table 2) [17].

### Statistical Analyses

All analyses were performed in the full MELODY enrollment cohort of 3012 infants. Data are presented descriptively. Incidence rates were calculated using the formula below, where events are defined as the first occurrence of an MA RSV LRTI and follow-up time is defined as the time elapsed from day of study drug administration to either the date of event occurrence or end of study for those who did not observe an event:


$$Incidence=\frac{Sum\;of\;\;events}{\mathrm{Follow}\text{-}\mathrm{up}\;\mathrm{time}\;(\mathrm{Days})}\times \frac{365.25\;Days}{1\;year}\times 100\;years$$

The 95% confidence intervals (CIs) for incidence rates were calculated using the formula:


$$\begin{array}{rl} 95\text{\%}\;CI & =\frac{100\;/\;\mathrm{Follow}\text{-}\mathrm{up}\;\mathrm{time}\;(\mathrm{Days})}{365.25}\\ & \;\times \left(Sum\;of\;\;events\;\pm 1.96\sqrt{\mathrm{Sum}\;\mathrm{of}\;\mathrm{events}}\right) \end{array}$$

Cumulative incidence was calculated per virus at each day on study by determining the cumulative sum of positive cases for that virus until that day, with days on study calculated as the difference between date of test and treatment start date; multiple episodes were considered in the calculation:


$$\mathrm{Cumulative}\;\mathrm{incidenc}e_{\mathrm{virus}}\mathrm{on}\;\mathrm{Day}\;X=\frac{\mathrm{Total}\;\mathrm{cases}\;\mathrm{of}\;\mathrm{virus}\;\mathrm{accured}\;\mathrm{to}\;\mathrm{Day}\;X}{\mathrm{Total}\;\mathrm{participants}\;\mathrm{in}\;\mathrm{arm}}\;\times 100$$

This analysis reports on the full study cohort through days 151, 361, and 511.

A proportional odds cumulative logit model was used to evaluate any differences in severity of infection between treatment groups. A proportional odds ratio and its 95% CI are reported. A Kappa analysis was conducted to characterize agreement for samples between BioFire and Lyra assays. A Cohen's kappa statistic, 95% CI, and nominal *P* value are reported. The kappa coefficient was interpreted using classifications published by Landis and Koch [18].

## RESULTS

### Participants and Samples

Baseline characteristics of the 561 participants with MA-LRTI who had nasopharyngeal swabs collected through day 511 (N = 852 swabs; 519 swabs from 337 nirsevimab-dosed participants and 333 swabs from 224 placebo-dosed participants) were generally similar between study arms (Supplementary Table 3). Supplementary Table 4 summarizes the number of swabs for each arm and time period.

### Pathogens Detected

Of the 852 nasopharyngeal swabs taken at the time of LRTI, 744 (87.3%) had viral infections detected via the BioFire Panel (Table 1), 451 of 519 in the nirsevimab arm and 293 of 333 in the placebo arm (Supplementary Tables 5 and 6). RSV infections were detected in 193 of 852 swabs (22.7%), wherein 126 of 193 (65.3%) were individual RSV infections and 67 of 193 (34.7%) were RSV coinfections. Among the 67 cases of RSV coinfections, there were 9 cases of RSV and VFAPn coinfection and 58 cases of RSV and ≥1 non-VFAPn coinfection, primarily rhinovirus/enterovirus with 43 unique cases. There were 24 cases of RT-PCR–confirmed RSV infection through day 151 in the nirsevimab arm and 54 in the placebo arm (Supplementary Table 7). Of these, 16 (69.6%) and 32 (66.7%) in each respective arm were single RSV infections per BioFire Panel analysis and 7 (30.4%) and 16 (33.3%) were coinfections with RSV and another pathogen, respectively; 1 case in the nirsevimab arm and 6 in the placebo arm were not available for analysis due to insufficient sample volumes. Supplementary Table 8 summarizes the number of swabs collected in each treatment arm for multiple LRTIs.

**Table 1.** Distribution of Types of Infection Detected via the BioFire Respiratory 2.1 Panel in 852 Nasopharyngeal Swabs Collected From 561 Participants With Medically Attended Lower Respiratory Tract Infection in the MELODY Study Pooled Across Arms, Day 1 Through Day 511

Type of Infection, n (%)	Total Number of Swabs (N = 852)	Individual Infection	Type of Coinfection							
			Any^a^	RV/ENT	Parainfluenza Virus 1–4	AdV	Seasonal hCoV	hMPV	Influenza Virus A/B	SARS-CoV-2
Any viral infection	744 (87.3)^b^	535 (62.8)^b^	209 (24.5)^b^	n/a	n/a	n/a	n/a	n/a	n/a	n/a
RSV	193 (22.7)^b^	126 (14.8)^b^	67 (7.9)^b^	46	7	16	9	2	1	4
Any non-RSV virus	551 (64.7)^b^	409 (48.0)^b^	142 (16.7)^b^	n/a	n/a	n/a	n/a	n/a	n/a	n/a
RV/ENT	404 (54.3)^c^	236	n/a	n/a	38	48	29	22	5	13
Parainfluenza virus (1–4)	124 (16.7)^c^	69	n/a	38	n/a	10	9	6	1	3
AdV	77 (10.3)^c^	12	n/a	48	10	n/a	6	2	1	2
Seasonal hCoV	76 (10.2)^c^	30	n/a	29	9	6	n/a	8	3	2
hMPV	63 (8.5)^c^	35	n/a	22	6	2	8	n/a	0	0
Influenza virus (A/B)	27 (3.6)^c^	18	n/a	5	1	1	3	0	n/a	0
SARS-CoV-2	29 (3.9)^c^	9	n/a	13	3	2	2	0	0	n/a
No pathogen detected	104 (12.2)^b^	n/a	n/a	n/a	n/a	n/a	n/a	n/a	n/a	n/a
Bacterial infection^d^	11 (1.3)^b^	4	7	5	0	0	1	1	3	0

Abbreviations: AdV, adenovirus; ENT, enterovirus; hCoV, human coronavirus; hMPV, human metapneumovirus; n/a, not applicable; RSV, respiratory syncytial virus; RV, rhinovirus; SARS-CoV-2, severe acute respiratory syndrome coronavirus 2.

^a^The sum of cases for each type of virus does not equal the total number of any cases due to coinfections.

^b^Percentage of total nasopharyngeal swabs; column percentages do not sum to 100% due to coinfections being seen in 209 swabs.

^c^Percentage of any viral infections.

^d^Any of *Bordetella pertussis*, *Bordetella parapertussis*, *Chlamydia pneumoniae*, *Mycoplasma pneumoniae* (note, *B. pertussis* infections were not detected in samples in this study).

Of the 142 cases of non-RSV coinfections detected (Table 1), 122 were rhinovirus/enterovirus (85.9%) infections. Episodes that comprised ≥2 non-RSV–VFAPn occurred in 6 of 142 (4.2%) cases, episodes with 1 non-RSV–VFAPn plus ≥1 non-VFAPn occurred in 75 of 142 (52.8%), and episodes with ≥2 non-VFAPn occurred in 66 of 142 (46.5%).

Eleven swabs had bacteria detected, precluding further analyses; viral coinfections were also detected among bacterial swabs. No pathogen was detected in 104 swabs. Overall, in 848 individual participants’ samples that were tested for RSV infection using both Lyra central laboratory RT-PCR and the BioFire Panel, almost perfect (kappa = 0.875) agreement was demonstrated for 95.9% (n = 813) of samples (Supplementary Table 9).

### Cumulative Incidence and Incidence Rates of Infections Detected Using the BioFire Panel

**Figure 1.**
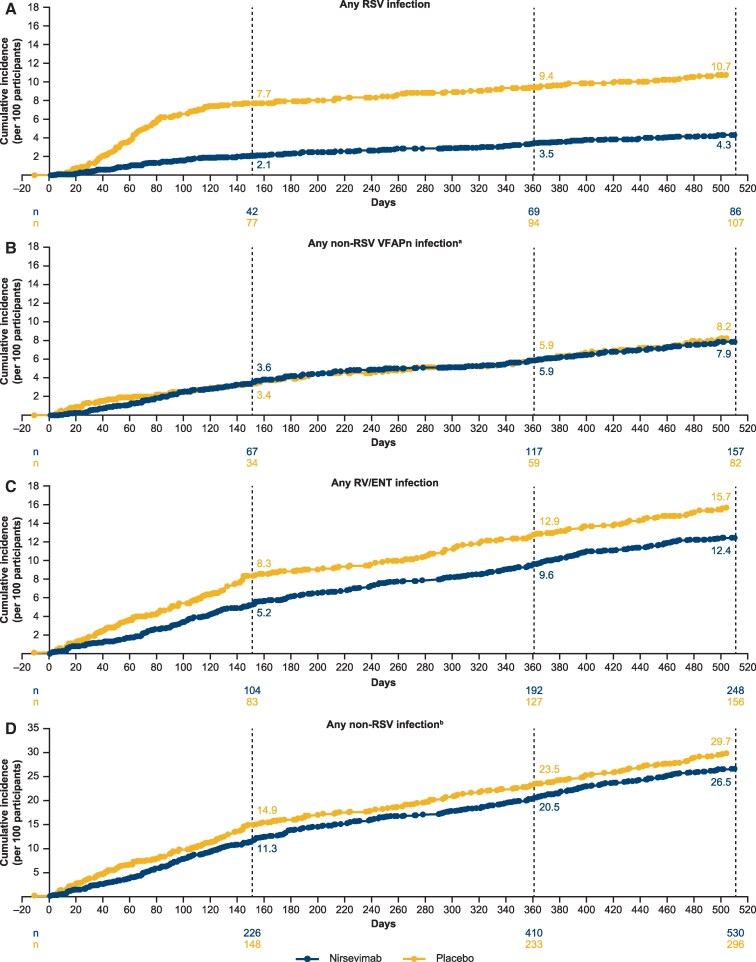
Cumulative incidence of RSV, non-RSV–VFAPn, RV/ENT, and all non-RSV infections detected via the BioFire Respiratory 2.1 Panel in participants in the nirsevimab and placebo arms across the whole study period (day 1 to day 511). Cumulative incidences are shown at days 151, 361, and 511. Coinfections are counted in all applicable individual cumulative incidence analyses. ^a^Analysis of any VFAPn infection is an additive analysis of cumulative incidence of human metapneumovirus, parainfluenza virus (1–4), and influenza virus (A/B) infections. ^b^Analysis of any non-RSV infection is an additive cumulative incidence of all infections except RSV. Cumulative incidence analyses include all new-onset events as detected using the BioFire panel within the respective categories of infection; that is, an individual with 2 separate infections with the same virus is counted twice in cumulative incidence analysis for that virus. Abbreviations: ENT, enterovirus; RSV, respiratory syncytial virus; RV, rhinovirus; VFAPn, viruses frequently associated with pneumonia.

The cumulative incidence of RSV infections from swabs collected due to suspected LRTI was lower with nirsevimab versus placebo at day 151 (Δ −5.6; Figure 1*A*); this difference increased slightly at day 361 (Δ −5.9) and day 511 (Δ −6.4). The cumulative incidence of non-RSV–VFAPn infections was similar between nirsevimab and placebo across the whole study (Figure 1*B*). The cumulative incidence of any parainfluenza infection was lower in the nirsevimab versus placebo group; the difference increased over the study period (day 511 Δ −1.4; Supplementary Figure 1A). The cumulative incidence of any influenza infection was similar between groups (day 511 Δ 0.0; Supplementary Figure 1B). There was a low but numerically higher cumulative incidence of hMPV infections in the nirsevimab versus placebo group, with a difference that increased over the study period (day 511 Δ +1.1; Supplementary Figure 1C).

The cumulative incidence of any rhinovirus/enterovirus infection was lower with nirsevimab versus placebo (Figure 1*C*) through day 151 (Δ −3.1) and remained similar at day 361 (Δ −3.3) and day 511 (Δ −3.3). The cumulative incidence of all non-RSV infections was numerically lower with nirsevimab versus placebo (Figure 1*D*). Minimal differences were seen between study arms in cumulative incidences of any AdV, seasonal hCOV, and SARS-CoV-2 infections (Supplementary Figures 1D–1F).

**Figure 2.**
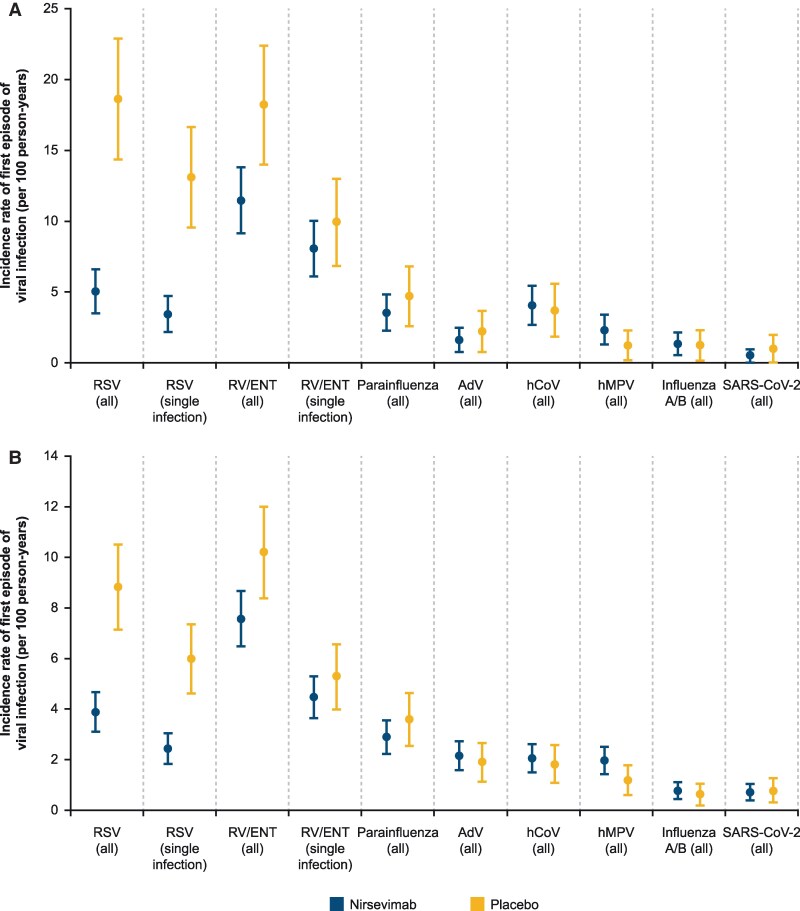
Incidence of first episodes of infection for each virus, regardless of type of infection (“All” = single infection or coinfection) or excluding coinfections (“Single infection”) for RSV and RV/ENT, through day 151 (*A*) and day 511 (*B*). Abbreviations: AdV, adenovirus; ENT, enterovirus; hCoV, human coronavirus; hMPV, human metapneumovirus; RSV, respiratory syncytial virus; RV, rhinovirus; SARS-CoV-2, severe acute respiratory syndrome coronavirus 2.

The incidence of infection per 100 person-years of at-risk infants through day 151 and day 511 are summarized in Figure 2. The incidence of any RSV and any rhinovirus/enterovirus infection was lower with nirsevimab versus placebo at both time points (albeit with overlapping CIs for any rhinovirus/enterovirus infection through day 511). To exclude the effect of coinfections, the incidence of single RSV or single rhinovirus/enterovirus infections was evaluated. There remained a statistically lower difference between nirsevimab and placebo groups for RSV incidence, but there was no difference between the groups for single rhinovirus/enterovirus incidence, with substantially overlapping CIs. The incidence of infections with other viruses was generally similar between groups at both time points.

**Figure 3.**
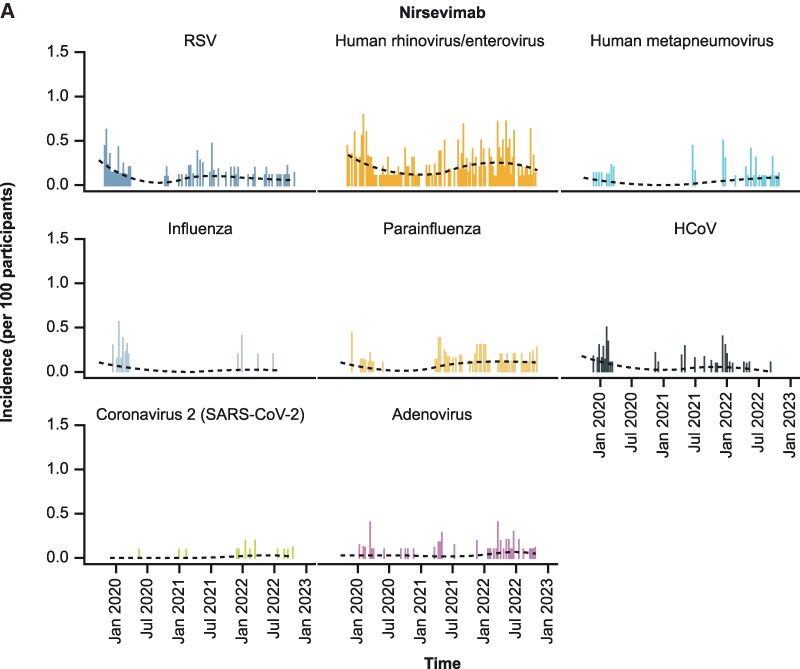

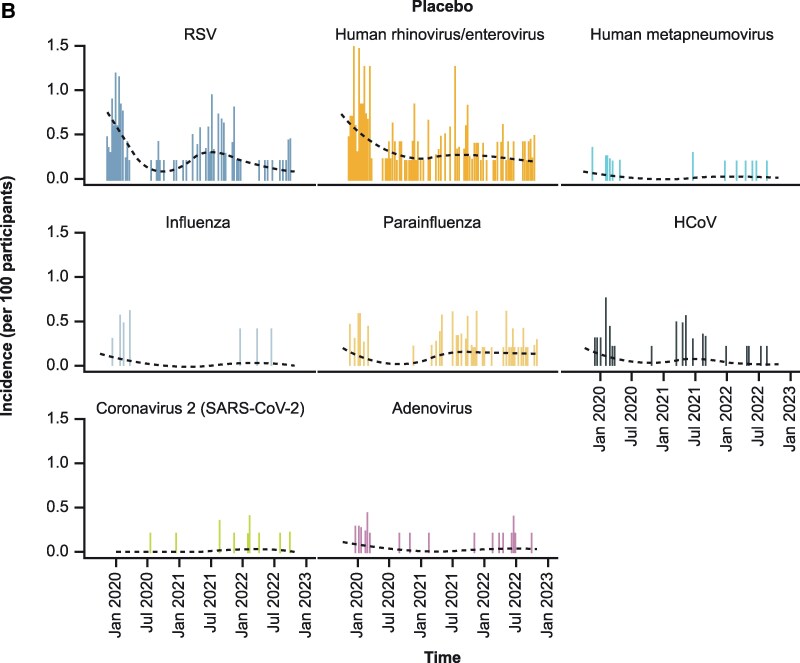
Incidence by calendar time of respiratory virus infections over the course of the 3-year MELODY study in participants in the nirsevimab (*A*) and placebo arms (*B*). Dashed line represents locally estimated scatterplot smoothing. Abbreviations: hCoV, human coronavirus; RSV, respiratory syncytial virus; SARS-CoV-2, severe acute respiratory syndrome coronavirus 2.

The incidence of respiratory virus infections over the 3-year study period reflected the disrupted seasonality of infections following nonpharmacologic COVID-19 pandemic control measures [19]. Furthermore, the incidence of RSV and rhinovirus/enterovirus infections was lower with nirsevimab versus placebo, both globally (Figure 3) and when stratified by hemisphere (Supplementary Figure 2).

### Severity of RSV Infections

**Figure 4.**
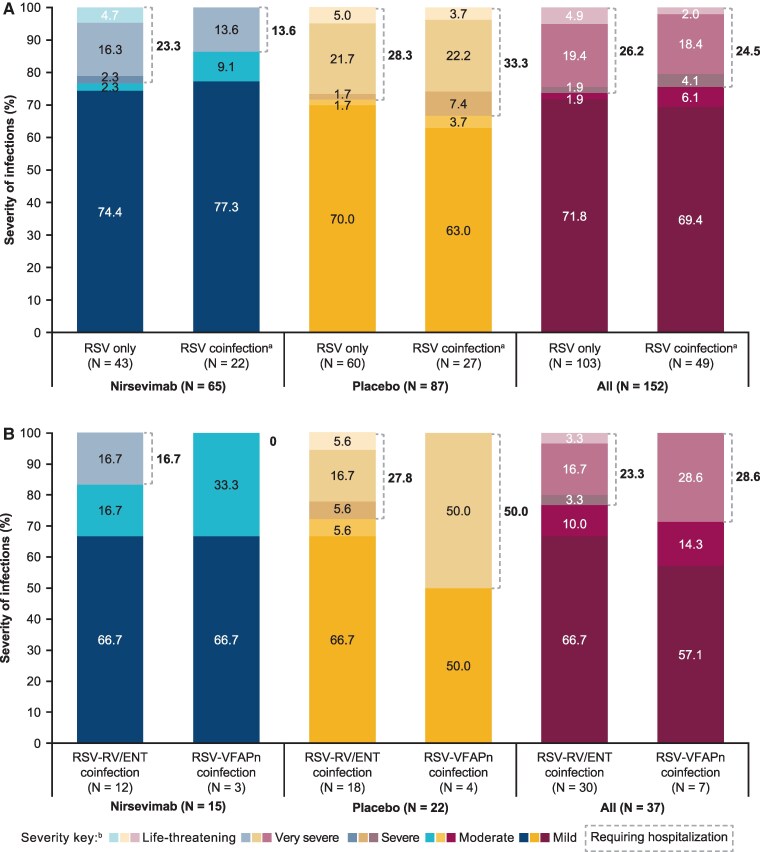
Severity of respiratory illness in RSV-associated medically attended lower respiratory tract infection through day 511 by study intervention and type of infection. *A*, RSV only or RSV with any coinfection. *B*, RSV with RV/ENT coinfection or RSV with any VFAPn coinfection. ^a^RSV coinfection includes coinfection with adenovirus, SARS-CoV-2, hCoV, human RV/ENT, and VFAPn (human metapneumovirus, influenza virus [A/B], parainfluenza virus [1–4]). ^b^Life-threatening = requiring respiratory support by mechanical ventilation/continuous positive airway pressure (CPAP)/high-flow nasal cannula (HFNC) or pediatric intensive care unit (PICU); very severe = hospitalized for RSV lower respiratory tract infection and requirement of oxygen or intravenous (IV) supply but not requiring mechanical ventilation/CPAP/HFNC or PICU; severe = hospitalized but not requiring oxygen or IV or mechanical ventilation/CPAP/HFNC/PICU (intensive care); moderate = ReSViNET score >6 but not hospitalized; mild = ReSViNET score ≤6 but not hospitalized [17]. Abbreviations: ENT, enterovirus; hCoV, seasonal human coronavirus; ReSViNET, Respiratory Syncytial Virus Network; RSV, respiratory syncytial virus; RV, rhinovirus; SARS-CoV-2, severe actute respiratory syndrme coronavirus 2; VFAPn, viruses frequently associated with pneumonia.

Data on the severity of RSV infections that occurred through day 511 were available for 152 of 744 cases (65 of 519 [12.5%] for nirsevimab, 87 of 333 [26.1%] for placebo). Overall, the severity of RSV infections was similar for single RSV infections and coinfections (*P* = .949; Figure 4*A*); however, there was a higher proportion of more severe cases in the placebo arm versus the nirsevimab arm (28.3% versus 23.3% RSV only, 33.3% versus 13.6% RSV coinfection). Approximately 70% of infections were classed as mild, and approximately one quarter required hospitalization. In the nirsevimab group, approximately three quarters of infections were classed as mild, and a numerically larger percentage of single RSV infections required hospitalization compared with RSV coinfections. There were similar percentages overall of RSV–rhinovirus/enterovirus coinfection and RSV–VFAPn coinfection that required hospitalization (Figure 4*B*). CT values were similar among participants irrespective of study intervention, single or coinfection, and by hospitalization (Supplementary Figure 3). Supplementary Figure 4 shows severity according to hospitalization or no hospitalization for non-RSV respiratory viruses.

## DISCUSSION

Nirsevimab prophylaxis led to a lower incidence of RSV infections over time versus placebo without viral replacement, that is, no increased incidence of other viral infections, consistent with efficacy observed against all-cause MA-LRTI (70.1%–76.4%) [7, 8, 12, 20]. The difference in cumulative MA-LRTI incidence primarily emerged during days 1–151. This difference between nirsevimab and placebo was maintained through day 511, with a slight numerical increase in the difference (the minimal difference driven primarily by cumulative incidence to day 151), coupled with a lower incident event rate of RSV infection with nirsevimab versus placebo to both day 151 and day 511. Data from South African participants in MELODY suggest nirsevimab efficacy beyond a typical 5-month RSV season [12]. Furthermore, a recent analysis of MELODY participants followed through their second RSV season showed that the incidence of RSV-associated MA-LRTI in the first (through day 151) and second (days 362–511) seasons was 1.7% and 1.8% with nirsevimab versus 7.5% and 2.1% with placebo [21]. Importantly, there was no increase in the incidence or severity of disease in the second season among infants who received nirsevimab in their first season relative to those who received placebo, suggesting no shift in the burden of disease and no evidence for antibody-dependent enhanced disease with nirsevimab [21, 22].

As expected, RSV, which was detected in approximately one quarter of samples, was one of the most common causative agents associated with LRTI, while rhinovirus/enterovirus, which was detected in more than half of samples, was the most common virus, including as a single-virus infection in more than a quarter of MA-LRTIs. The contribution of rhinovirus to LRTI is debated, and rhinoviruses have been detected in participants with and without LRTI [23]. Recent findings show a consistent prevalence of rhinovirus (and AdV/hCoV) infections among infants with community-acquired alveolar pneumonia or bronchiolitis [23, 24]. Additionally, rhinovirus has been associated with cases of pneumonia in hospitalized children aged 12–59 months in case-control studies and is frequently detected in community controls and children who undergo elective surgery [25, 26]. In our study, the apparently lower cumulative incidence and incidence rate of rhinovirus/enterovirus infections in the nirsevimab versus placebo group was driven by the lower rate of detection as a coinfection with RSV. Previous analyses have shown that rhinovirus is detected in up to two thirds of RSV-related hospitalizations before nirsevimab implementation [14]. After rhinovirus/enterovirus-only coinfections that may not be contributing to disease are removed, the cumulative incidence and incidence rate of rhinovirus/enterovirus-only infections were similar in nirsevimab and placebo recipients, suggesting that rhinovirus/enterovirus infection does not replace RSV in contributing to LRTI.

The cumulative incidence and incidence rate of influenza virus (A/B) detected were low and similar between groups, which is expected given the generally lower rates of influenza in this age group compared with RSV. The cumulative incidence of any non-RSV–VFAPn over time was similar between groups, while cumulative incidence and incidence rates of hMPV infections over time were numerically higher in the nirsevimab versus placebo groups, albeit at very low levels. Although the low incidence rates limit interpretation, further surveillance from real-world settings and analyses of studies that used monoclonal antibodies are needed to consider potential replacement of RSV with hMPV, particularly as these viruses are both part of the *Pneumoviridae* subfamily [27].

Before the COVID-19 pandemic, respiratory viruses followed regular seasonal trends, with RSV being epidemic over winter in temperate climates [4]. Interventions initiated to control the spread of SARS-CoV-2 disrupted the seasonal patterns of endemic respiratory viruses [28–30]. Compared with the pre-pandemic period, RSV incidence and hospitalizations in temperate climates were lower during the winter months of 2020, followed by a surge in cases during spring/summer [23, 28–30]. Although some studies suggest no change in the severity of illness compared with the pre-pandemic period, with similar levels of intensive care unit (ICU) admission, length of stay, and case fatalities in 2021–2022 [31], these findings are inconclusive. In some regions, reduced RSV circulation was accompanied by a notable shift in the viral landscape, as evidenced by a sudden and substantial increase in rhinovirus prevalence [32]. Our data also show a disrupted respiratory virus seasonality. Importantly, following the administration of nirsevimab, there was no replacement of RSV with other viruses, including rhinovirus/enterovirus, during the study period. The mechanism(s) through which viral replacement was prevented remains unknown. It has been hypothesized that the interferon response following the first viral infection confers a temporary nonspecific immunity against infection by other viruses [33]. Conversely, infection by a first virus could enhance infection and replication of a second virus through positive interaction [33]; therefore, removal of the first virus (by nirsevimab in the case of RSV) may contribute to prevention of viral replacement.

Overall, severity of infections was broadly similar between single RSV infections and coinfections. Previous studies have suggested that, despite the high prevalence of RSV coinfection, this was generally not associated with increased clinical severity (risk of fever, hospitalization or ICU admission, oxygen use, ventilation, and death) compared with single viral infection [11, 34–36]. The rate of hospitalization was numerically higher with single RSV infections than with RSV coinfections in the nirsevimab group and higher in the placebo versus nirsevimab group, suggesting that RSV is a significant driver of severe disease following an infection in this population. Previous analyses of nirsevimab have shown no enhanced disease severity relative to placebo [20, 21]. Similar findings were observed in a 3-month analysis of the ongoing 3-year evaluation of the effectiveness and impact of Nirsevimab in Galicia (NIRSE-GAL) study in a real-world setting. The effectiveness of nirsevimab was 82.0% against RSV-related LRTI hospitalizations, 86.9% against severe RSV-related LRTI requiring oxygen, and 69.2% against all-cause LRTI hospitalizations [13], consistent with observations from smaller studies [37, 38].

Our study has some limitations. Importantly, like all diagnostic tests, the BioFire^®^ Panel may return negative results despite an LRTI, which may be due to pathogens not detected by the test or the timing of nasopharyngeal swab sampling failing to detect a pathogen associated with disease [15]. Consequently, negative results do not preclude infection. Furthermore, viral nucleic acids may persist *in vivo*, and detection does not imply organisms that are infectious or causative for clinical symptoms. These occurrences highlight the importance of clinical observations, patient history, and epidemiological information. The BioFire^®^ Panel is highly sensitive, although direct comparisons with other assays are limited by the isolate source material used. The analyses conducted herein were not powered to detect statistical differences; rather, CIs have been used for inference and interpretation. Although the study included nasopharyngeal swabs taken from many participants with MA-LRTI, very low incidence rates and small numbers of some coinfections detected, such as RSV-VFAPn coinfections, limit the interpretation of findings and the utility of further analyses, including stratification by time period or by hemisphere (for seasonality). While our surveillance was continuous over 511 days and included 2 RSV seasons in most regions, the follow-up did not extend for a full second year and does not preclude the possibility that respiratory viruses continued to circulate and may have given rise to events subsequently. The use of a broad multiplex PCR panel could result in decreased sensitivity and detection of viruses other than RSV and hMPV; however, multiplex panels are widely used and generally considered highly sensitive [39, 40]. Furthermore, the multiplex PCR panel does not differentiate between enteroviruses and rhinoviruses.

In conclusion, the lower incidence rates of RSV infection in nirsevimab recipients versus placebo, coupled with similar incidences of non-RSV respiratory viruses between nirsevimab and placebo participants, further support the nirsevimab-specific prevention of RSV and suggest there is no replacement of RSV with other viruses following nirsevimab administration.

## Supplementary Material

ciae596_Supplementary_Data

## Supplementary Data

Supplementary materials are available at *Clinical Infectious Diseases* online. Consisting of data provided by the authors to benefit the reader, the posted materials are not copyedited and are the sole responsibility of the authors, so questions or comments should be addressed to the corresponding author.

## Notes

***Author contributions.*** Conceptualization: A. A. A., D. A., D. W., E. J. K., M. T. E., R. D., T. L. V., X. S. L. Data curation: A. A. A., D. W., V. G. Formal analysis: A. A. A., A. L., D. A., R. D., V. G., X. S. L., Y. C. Funding acquisition: M. T. E., T. L. V. Investigation: E. J. K., M. T. E., R. D., V. S. M., X. S. L. Methodology: D. A., D. W., E. J. K., L. L. H., X. S. L. Project administration: A. A. A. Resources: M. T. E., T. L. V. Supervision: E. J. K., L. L. H., X. S. L. Validation: B. A., D. A., D. S., V. G., X. S. L. Visualization: A. L., D. A., D. W., V. G., X. S. L. Writing the original draft: A. A. A., D. W., V. G. Writing, reviewing, and editing: A. A. A., A. L., B. A., D. A., D. S., D. W., E. J. K., L. L. H., M. T. E., R. D., T. L. V., V. G., V. S. M., Y. C., X. S. L.

***Acknowledgments.*** Medical writing support for the development of this manuscript, under the direction and guidance of the authors, was provided by Paul Barlass, PhD, and Craig O’Hare, PhD, of Ashfield MedComms, an Inizio Company, in accordance with Good Publications Practice guidelines (https://www.ismpp.org/gpp-2022). This support was funded by AstraZeneca.

***Disclaimer.*** The study sponsors participated in the study design and managed all operational aspects of the study, including data collection, analysis and interpretation, preparation of the manuscript, and decision to submit the manuscript for publication.

***Financial support*.** The MELODY trial was funded by AstraZeneca United States and Sanofi United States. Nirsevimab is being developed and commercialized in partnership between AstraZeneca and Sanofi.

***Data sharing statement.*** Data that underlie the findings described here may be obtained in accordance with AstraZeneca's data sharing policy described at https://astrazenecagrouptrials.pharmacm.com/ST/Submission/Disclosure. Data for studies directly listed on Vivli can be requested through Vivli at www.vivli.org. Data for studies not listed on Vivli can be requested through Vivli at https://vivli.org/members/enquiries-about-studies-not-listed-on-the-vivli-platform/. The AstraZeneca Vivli member page is also available and outlines further details: https://vivli.org/ourmember/astrazeneca/.
