# Supplementary material for: Lower Respiratory Tract Infections Following Respiratory Syncytial Virus Monoclonal Antibody Nirsevimab Immunization Versus Placebo: Analysis From a Phase 3 Randomized Clinical Trial (MELODY)
Source: Clin Infect Dis. 2024 Dec 4;81(3):634–44. doi: 10.1093/cid/ciae596 (PMC12497957; doi:10.1093/cid/ciae596)
Supplement: ciae596_Supplementary_Data [file ciae596_supplementary_data.docx]

**Supplementary Data to Arbetter D., et al.**

**Lower respiratory tract infections following respiratory syncytial virus monoclonal antibody nirsevimab immunization versus placebo: Analysis from a Phase 3 randomized clinical trial (MELODY)**

**Table of Contents**

[Supplemental Table 1*.* Criteria for diagnosis of MA LRTI. 2](#_Toc179903266)

[Supplemental Table 2. ReSVinet scale used to define LRTI severity [1]. 3](#_Toc179903267)

[Supplementary Table 3. Demographics and baseline characteristics of participants from which nasopharyngeal swabs were collected. 4](#_Toc179903268)

[Supplementary Table 4. Numbers of participants from which nasopharyngeal swabs were collected overall and in each period. 5](#_Toc179903269)

[Supplementary Table 5. Distribution of types of infection detected via the BioFire^®^ Respiratory 2.1 Panel in 519 nasopharyngeal swabs collected from 337 participants in MELODY, Day 1 through Day 511 – nirsevimab arm. 6](#_Toc179903270)

[Supplementary Table 6. Distribution of types of infection detected via the BioFire^®^ Respiratory 2.1 Panel in 333 nasopharyngeal swabs collected from 224 participants in MELODY, Day 1 through Day 511 – placebo arm. 7](#_Toc179903271)

[Supplementary Table 7. Cases of RSV infection (RT-PCR-confirmed, per primary case definition) and coinfection status in participants in the nirsevimab and placebo arms, Day 1 through Day 151. 9](#_Toc179903272)

[Supplementary Table 8. Numbers of nasopharyngeal swabs collected and percentage of cases with multiple LRTIs in each treatment arm. 9](#_Toc179903273)

[Supplementary Table 9. Concordance between RSV testing per Lyra^®^ central laboratory RT-PCR and per BioFire^®^ Respiratory 2.1 Panel. 10](#_Toc179903274)

[Supplementary Figure 1. Cumulative incidence of any parainfluenza virus (1–4), influenza virus (A/B), hMPV, AdV, seasonal hCoV, and SARS-CoV-2 infections as detected via the BioFire Respiratory 2.1 Panel in participants in the nirsevimab and placebo arms across the whole study period (Days 1–511). 11](#_Toc179903275)

[Supplementary Figure 2. Incidence by calendar time of respiratory virus infections over the course of the 3-year MELODY study in the northern and southern hemispheres in participants in the nirsevimab (A and B) and placebo (C and D) arms. 14](#_Toc179903276)

[Supplementary Figure 3. Viral loads for participants with MA RSV LRTI (protocol defined) through Day 511 according to RSV coinfection status (A) and hospitalization status (B and C). 16](#_Toc179903277)

[Supplementary Figure 4. Severity of all MA LRTIs (any cause) by treatment group according to hospitalization or no hospitalization for non-RSV respiratory viruses. 17](#_Toc179903278)

## Supplemental Table 1*.* Criteria for diagnosis of MA LRTI.

| MA-LRTI | Etiology | Lower respiratory tract | Medical significance |
| --- | --- | --- | --- |
| MA RSV LRTI (Primary case definition) | RSV confirmed positive by central laboratory real-time RT-PCR assay | Documented physical examination findings localizing to lower respiratory tract:   - Rhonchi - Rales - Crackles - Wheeze | At least one of the following clinical signs:   - Increased respiratory rate rest (aged <2 months, ≥60 breaths/min; aged 2–6 months, ≥50 breaths/min; aged >6 months, ≥40 breaths/min) - Hypoxemia (in room air: O_2_ saturation <95% at altitudes ≤1,800 meters or <92% at altitudes >1,800 meters) - Clinical signs of severe respiratory disease:   - Acute hypoxic or ventilatory failure   - New onset apnea   - Nasal flaring   - Retractions (intercostal, subcostal, or supraclavicular)   - Grunting - Dehydration due to respiratory distress |
| Non-RSV LRTI | Presence of non-RSV pathogen detected by BioFire^®^ 2.1 Respiratory Panel |  |  |

One item from the etiology, lower respiratory tract, and medical significance columns is required to meet the definition of MA-LRTI.

Abbreviations: MA LRTI, medically attended lower respiratory tract infection; RSV, respiratory syncytial virus; RT-PCR, reverse transcription polymerase chain reaction.

## Supplemental Table 2. ReSVinet scale used to define LRTI severity [1].

| Severity | Definition |
| --- | --- |
| Mild | ReSVinet score ≤6, but not hospitalized |
| Moderate | ReSVinet score >6, but not hospitalized |
| Severe | Hospitalized, but not requiring oxygen or IV supply or mechanical ventilation/CPAP/HFNC/PICU; intensive care |
| Very Severe | Hospitalized for RSV LRTI and requirement of oxygen or IV supply but not requiring mechanical ventilation/CPAP/HFNC or PICU |
| Life-threatening | Requiring respiratory support by mechanical ventilation/CPAP/HFNC or PICU |

Abbreviations: CPAP, continuous positive airway pressure; HFNC, High-flow nasal cannula; IV, intravenous, LRTI, lower respiratory tract infection; PICU, pediatric intensive care unit admission; RSV, respiratory syncytial virus.

1. Justicia-Grande AJ, Pardo-Seco J, Cebey-Lopez M, et al. Development and Validation of a New Clinical Scale for Infants with Acute Respiratory Infection: The ReSVinet Scale. PLoS One **2016**; 11: e0157665.

## Supplementary Table 3. Demographics and baseline characteristics of participants from which nasopharyngeal swabs were collected.

| Characteristic | Nirsevimab (N = 337) | Placebo (N = 224) | Total (N = 561) |
| --- | --- | --- | --- |
| Age, months; median (min, max) | 2.27 (0.03–9.36) | 2.33 (0.03–11.01) | 2.27 (0.03–11.01) |
| Sex, n (%) |  |  |  |
| Female | 134 (39.8) | 97 (43.3) | 231 (41.2) |
| Race, n (%) |  |  |  |
| American Indian or Alaska Native | 16 (4.7) | 15 (6.7) | 31 (5.5) |
| Asian | 22 (6.5) | 14 (6.3) | 36 (6.4) |
| Black or African American | 52 (15.4) | 38 (17.0) | 90 (16.0) |
| Native Hawaiian or other Pacific Islander | 1 (0.3) | 2 (0.9) | 3 (0.5) |
| White | 176 (52.2) | 109 (48.7) | 285 (50.8) |
| Other or multiple categories | 70 (20.8) | 46 (20.5) | 116 (20.7) |
| Weight, kg; median (min, max) | 5.2 (2.0–10.8) | 5.7 (2.0–11.0) | 5.4 (2.0–11.0) |
| Weight group on Day 1, n (%) |  |  |  |
| <2.5 kg | 8 (2.4) | 6 (2.7) | 14 (2.5) |
| <5 kg | 150 (44.5) | 87 (38.8) | 237 (42.2) |
| ≥5 kg | 187 (55.5) | 137 (61.2) | 324 (57.8) |
| Gestational age group, n (%) |  |  |  |
| ≥35 weeks to <37 weeks | 51 (15.1) | 31 (13.8) | 82 (14.6) |
| ≥37 weeks | 286 (84.9) | 193 (86.2) | 479 (85.4) |

Race was reported by parents/guardians; each category includes participants where only that category was selected while “Other or multiple categories” refers to a category other than those listed or for whom more than one category was checked.

## Supplementary Table 4. Numbers of participants from which nasopharyngeal swabs were collected overall and in each period.

|  | Nirsevimab | | Placebo | | Total | |
| --- | --- | --- | --- | --- | --- | --- |
|  | Swabs | Participants^a^ | Swabs | Participants^a^ | Swabs | Participants |
| Total | 519 | 337 | 333 | 224 | 852 | 561 |
| Day 1–151 | 249 | 203 | 185 | 148 | 434 | 351 |
| Day 152–361 | 155 | 122 | 87 | 66 | 242 | 188 |
| Day 362–511 | 115 | 89 | 61 | 50 | 176 | 139 |

^a^Total unique nasopharyngeal swabs obtained from 501 unique participants; participants can be counted in more than one period (i.e., Day 1–151, 152–361, 361–511) as a participant could experience multiple LRTIs and thus submit more than one swab throughout the study.

LRTI, lower respiratory tract infection.

## Supplementary Table 5. Distribution of types of infection detected via the BioFire^®^ Respiratory 2.1 Panel in 519 nasopharyngeal swabs collected from 337 participants in MELODY, Day 1 through Day 511 – nirsevimab arm.

| Type of infection, n (%) | TOTAL swabs  (N = 519) | Individual infection | Type of coinfection | | | | | | | |
| --- | --- | --- | --- | --- | --- | --- | --- | --- | --- | --- |
|  |  |  | Any^d^ | RV/ENT | Parainfluenza virus 1–4 | AdV | Seasonal hCoV | hMPV | Influenza virus A/B | SARS-CoV-2 |
| Any viral infection | 451 (86.9)^a^ | 329 (63.4)^a^ | 122 (23.5)^a^ | n/a | n/a | n/a | n/a | n/a | n/a | n/a |
| RSV | 86 (16.6)^a^ | 57 (11.0)^a^ | 29 (5.6)^a^ | 19 | 3 | 9 | 4 | 0 | 1 | 1 |
| Any non-RSV virus | 365 (70.3)^a^ | 272 (52.4)^a^ | 93 (17.9)^a^ | n/a | n/a | n/a | n/a | n/a | n/a | n/a |
| RV/ENT | 248 (55.0)^b^ | 148 | n/a | n/a | 19 | 36 | 20 | 17 | 3 | 9 |
| Parainfluenza virus (1–4) | 73 (16.2)^b^ | 44 | n/a | 19 | n/a | 6 | 7 | 5 | 1 | 2 |
| AdV | 54 (12.0)^b^ | 10 | n/a | 36 | 6 | n/a | 3 | 0 | 0 | 2 |
| Seasonal hCoV | 51 (11.3)^b^ | 22 | n/a | 20 | 7 | 3 | n/a | 6 | 3 | 1 |
| hMPV | 49 (10.9)^b^ | 29 | n/a | 17 | 5 | 0 | 6 | n/a | 0 | 0 |
| Influenza virus (A/B) | 19 (4.2)^b^ | 13 | n/a | 3 | 1 | 0 | 3 | 0 | n/a | 0 |
| SARS-CoV-2 | 19 (4.2)^b^ | 6 | n/a | 9 | 2 | 2 | 1 | 0 | 0 | n/a |
| No pathogen detected | 39 (7.5)^a^ | n/a | n/a | n/a | n/a | n/a | n/a | n/a | n/a | n/a |
| Bacterial infection only^c^ | 6 (1.2)^a^ | 3 | 3 | 2 | 0 | 0 | 1 | 0 | 0 | 0 |

^a^Percentage of total nasopharyngeal swabs; column percentages do not sum to 100% due to coinfections being seen in 209 swabs. ^b^Percentage of any viral infections. ^c^Any of *Bordetella pertussis*, *B. parapertussis*, *Chlamydia pneumoniae*, *Mycoplasma pneumoniae* (note, *B. pertussis* infections were not detected in samples in this study). ^d^The sum of cases for each type of virus does not equal the total number of any cases due to coinfections.

Abbreviations: AdV, adenovirus; ENT, enterovirus; hCoV, human coronavirus; hMPV, human metapneumovirus; n/a, not applicable; RSV, respiratory syncytial virus; RV, rhinovirus; SARS-CoV-2, severe acute respiratory syndrome coronavirus 2.

## Supplementary Table 6. Distribution of types of infection detected via the BioFire^®^ Respiratory 2.1 Panel in 333 nasopharyngeal swabs collected from 224 participants in MELODY, Day 1 through Day 511 – placebo arm.

| Type of infection, n (%) | TOTAL swabs  (N = 333) | Individual infection | Type of coinfection | | | | | | | |
| --- | --- | --- | --- | --- | --- | --- | --- | --- | --- | --- |
|  |  |  | Any^d^ | RV/ENT | Parainfluenza virus 1–4 | AdV | Seasonal hCoV | hMPV | Influenza virus A/B | SARS-CoV-2 |
| Any viral infection | 293 (88.0)^a^ | 206 (61.9)^a^ | 87 (26.1)^a^ | n/a | n/a | n/a | n/a | n/a | n/a | n/a |
| RSV | 107 (32.1)^a^ | 69 (20.7)^a^ | 38 (11.4)^a^ | 27 | 4 | 7 | 5 | 2 | 0 | 3 |
| Any non-RSV virus | 186 (55.9)^a^ | 137 (41.1)^a^ | 49 (14.7)^a^ | n/a | n/a | n/a | n/a | n/a | n/a | n/a |
| RV/ENT | 156 (53.2)^b^ | 88 | n/a | n/a | 19 | 12 | 9 | 5 | 2 | 4 |
| Parainfluenza virus (1­–4) | 51 (17.4)^b^ | 25 | n/a | 19 | n/a | 4 | 2 | 1 | 0 | 1 |
| AdV | 23 (7.8)^b^ | 2 | n/a | 12 | 4 | n/a | 3 | 2 | 1 | 0 |
| Seasonal hCoV | 25 (8.5)^b^ | 8 | n/a | 9 | 2 | 3 | n/a | 2 | 0 | 1 |
| hMPV | 14 (4.8)^b^ | 6 | n/a | 5 | 1 | 2 | 2 | n/a | 0 | 0 |
| Influenza virus (A/B) | 8 (2.7)^b^ | 5 | n/a | 2 | 0 | 1 | 0 | 0 | n/a | 0 |
| SARS-CoV-2 | 10 (3.4)^b^ | 3 | n/a | 4 | 1 | 0 | 1 | 0 | 0 | n/a |
| No pathogen detected | 39 (11.7)^a^ | n/a | n/a | n/a | n/a | n/a | n/a | n/a | n/a | n/a |
| Bacterial infection only^c^ | 5 (1.5)^a^ | 1 | 4 | 3 | 0 | 0 | 0 | 1 | 3 | 0 |

^a^Percentage of total nasopharyngeal swabs; column percentages do not sum to 100% due to coinfections being seen in 209 swabs. ^b^Percentage of any viral infections. ^c^Any of *Bordetella pertussis*, *B. parapertussis*, *Chlamydia pneumoniae*, *Mycoplasma pneumoniae* (note, *B. pertussis* infections were not detected in samples in this study). ^d^The sum of cases for each type of virus does not equal the total number of any cases due to coinfections.

Abbreviations: AdV, adenovirus; ENT, enterovirus; hCoV, human coronavirus; hMPV, human metapneumovirus; n/a, not applicable; RSV, respiratory syncytial virus; RV, rhinovirus; SARS-CoV-2, severe acute respiratory syndrome coronavirus 2.

## Supplementary Table 7. Cases of RSV infection (RT-PCR-confirmed, per primary case definition) and coinfection status in participants in the nirsevimab and placebo arms, Day 1 through Day 151.

| Type of infection, n (%) | Nirsevimab (N = 2009) | Placebo (N = 1003) |
| --- | --- | --- |
| Medically attended RSV-associated infection | n = 24 (1.2) | n = 54 (5.4) |
| Coinfection status | 23 (95.8) | 48 (88.9) |
| RSV alone, no coinfection | 16 (69.6) | 32 (66.7) |
| RSV with coinfection | 7 (30.4) | 16 (33.3) |
| Not available^a^ | 1 | 6 |

^a^Cases of RT-PCR-confirmed RSV infection in which sample for analysis by BioFire^®^ Respiratory 2.1 Panel not available due to insufficient sample volumes.

Abbreviations: RSV, respiratory syncytial virus; RT-PCR, reverse transcriptase polymerase chain reaction.

## Supplementary Table 8. Numbers of nasopharyngeal swabs collected and percentage of cases with multiple LRTIs in each treatment arm.

| Nirsevimab | | | Placebo | | |
| --- | --- | --- | --- | --- | --- |
| LRTI number | Count | % of all cases with multiple episodes | LRTI number | Count | % of all cases with multiple episodes |
| 2 | 92 | 54.76 | 2 | 50 | 29.76 |
| 3 | 32 | 19.05 | 3 | 18 | 10.71 |
| 4 | 11 | 6.55 | 4 | 6 | 3.57 |
| 5 | 3 | 1.79 | 5 | 1 | 0.6 |
| 6 | 3 | 1.79 | – | – | – |
| 7 | 1 | 0.6 | – | – | – |

Participants were required to have at least a 30-day interval between successive episodes. Total number of participants in both arms who had multiple LRTIs was 168. Through 150 days post-dose, only 15 participants in the placebo group and 21 participants in the nirsevimab group had BioFire data for a second LRTI.

## Supplementary Table 9. Concordance between RSV testing per Lyra^®^ central laboratory RT-PCR and per BioFire^®^ Respiratory 2.1 Panel.

| Data shown as n (%) of total number of individual participants’ samples tested by both methods | | Central laboratory RT-PCR RSV status | | Total |
| --- | --- | --- | --- | --- |
|  |  | Lyra^®^ | |  |
|  |  | Detected | Not detected |  |
| BioFire^®^ RSV status | Detected | 159 (82.8) | 33 (17.2) | 192 (22.6) |
|  | Not detected | 2 (0.3) | 654 (99.7) | 656 (77.4) |
| Total | | 161 (19.0) | 687 (81.0) | 848 (100) |
| Overall concordance | | | | 813 (95.9) |
| Kappa coefficient (SE)  [95% confidence limits] | | | | 0.875 (0.0205)  [0.8348–0.9153] |

Abbreviations: RSV, respiratory syncytial virus; RT-PCR, reverse transcriptase polymerase chain reaction; SE, standard error.

## Supplementary Figure 1. Cumulative incidence of any parainfluenza virus (1–4), influenza virus (A/B), hMPV, AdV, seasonal hCoV, and SARS-CoV-2 infections as detected via the BioFire Respiratory 2.1 Panel in participants in the nirsevimab and placebo arms across the whole study period (Days 1–511).


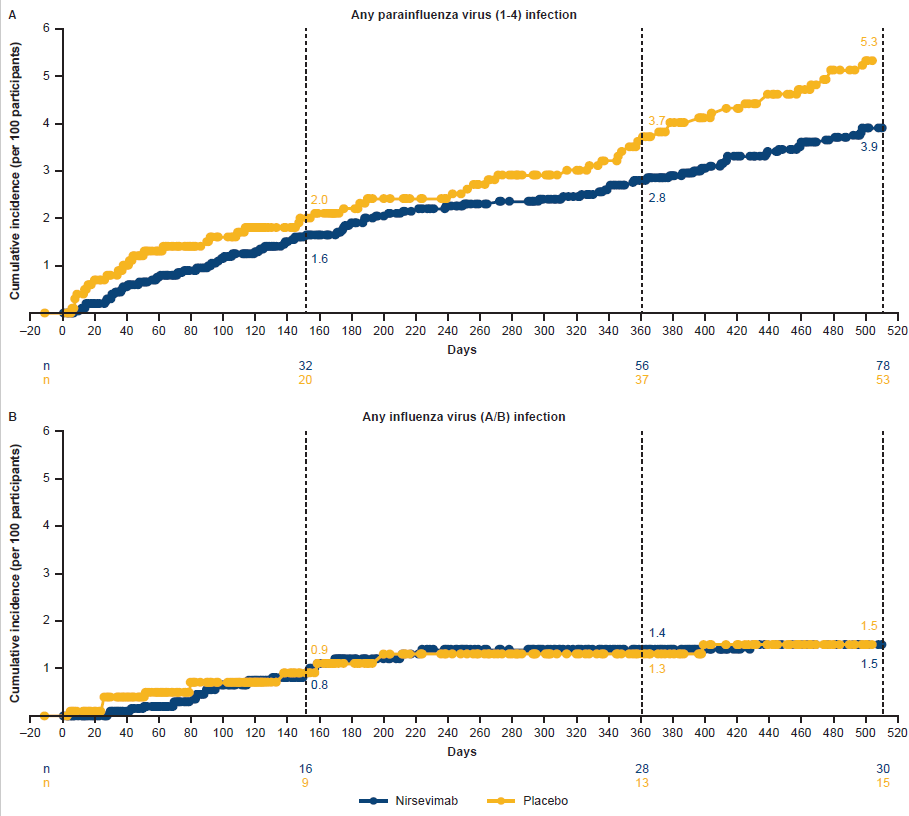


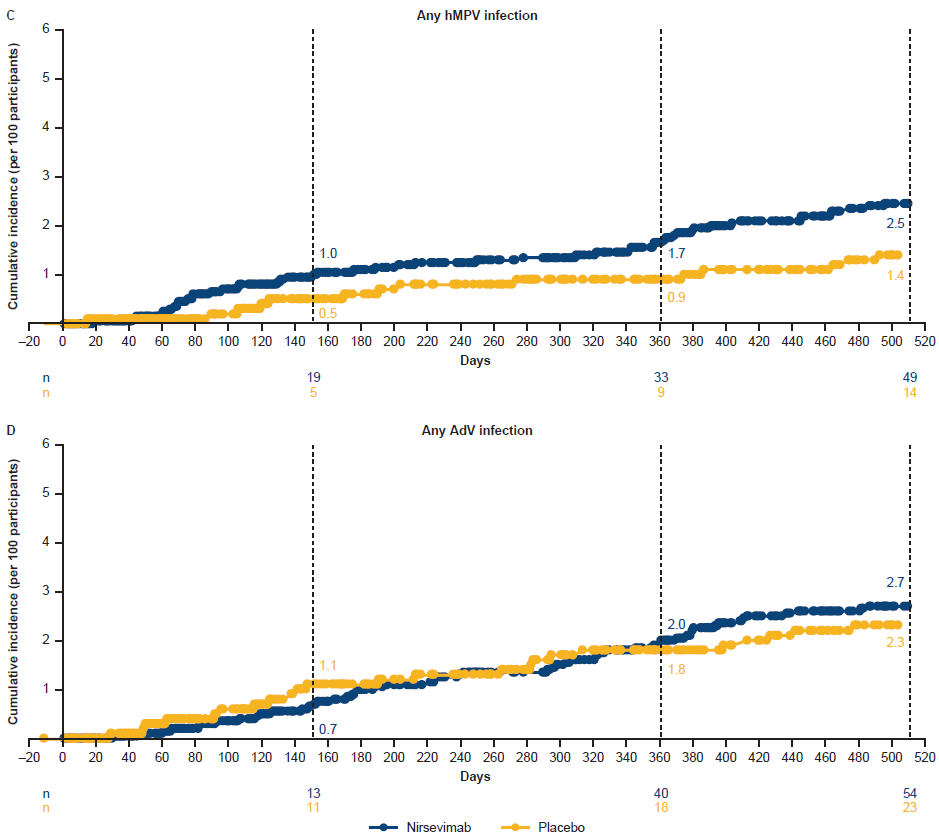


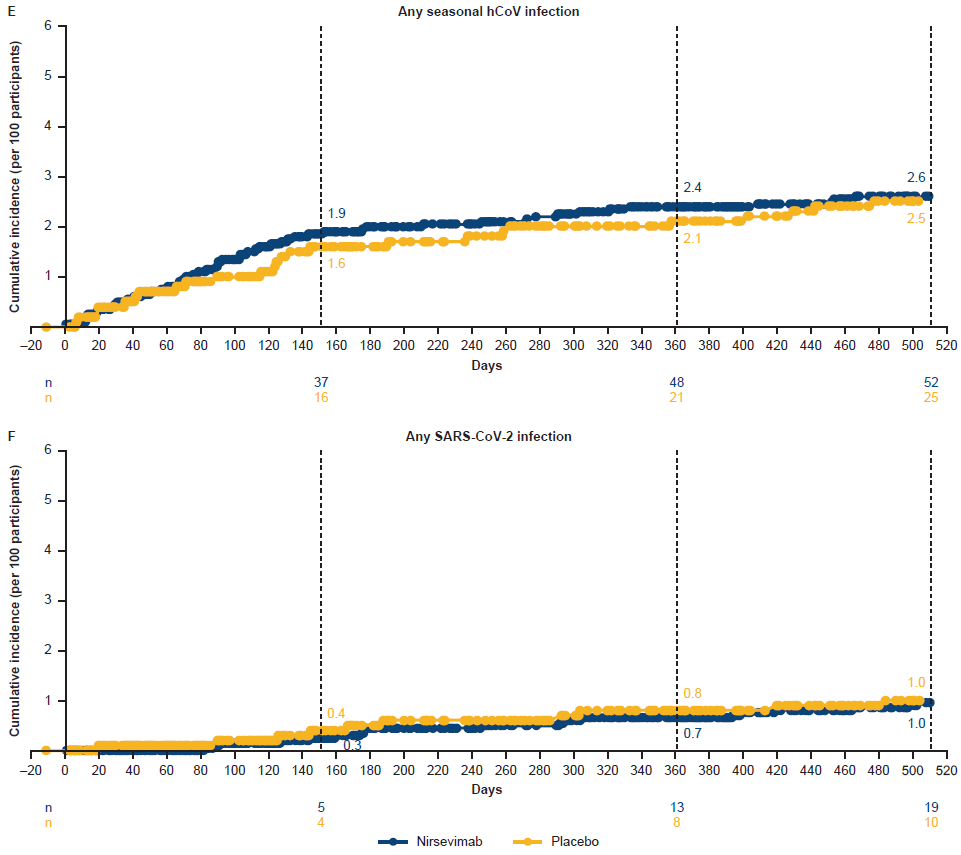


Cumulative incidences are shown at Days 151, 361, and 511. Coinfections are counted in all applicable individual cumulative incidence analyses.

Cumulative incidence analyses include all protocol-defined new-onset events within the respective categories of infection; i.e. an individual with two separate infections with the same virus is counted twice in the cumulative incidence analysis for that virus.

Abbreviations: AdV, adenovirus; hCoV, human coronavirus; hMPV, human metapneumovirus; SARS-CoV-2, severe acute respiratory syndrome coronavirus 2.

## Supplementary Figure 2. Incidence by calendar time of respiratory virus infections over the course of the 3-year MELODY study in the northern and southern hemispheres in participants in the nirsevimab (A and B) and placebo (C and D) arms.


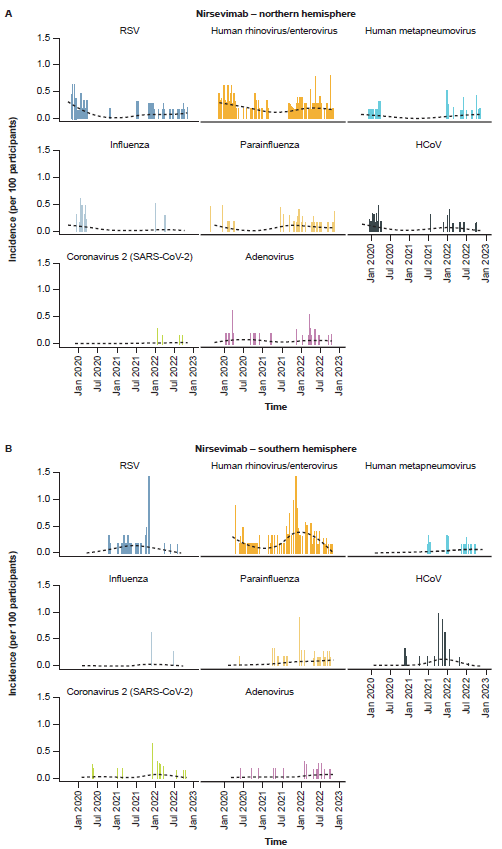


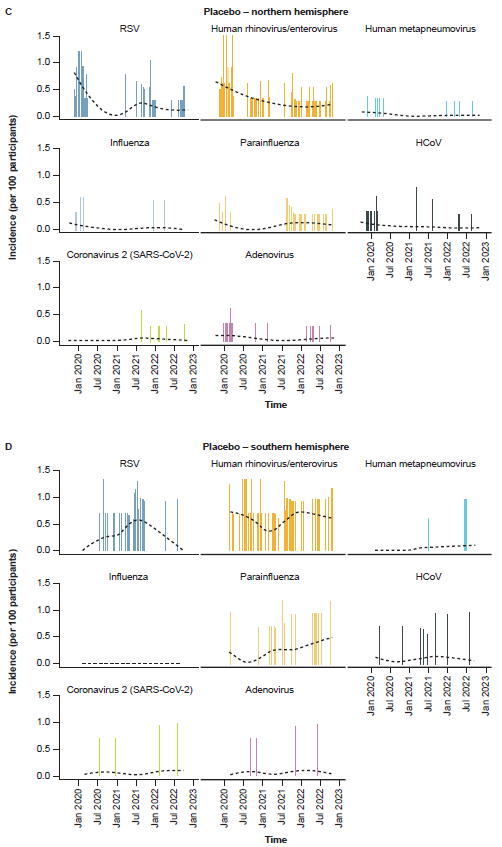


Dashed line represents LOESS smoothing.
Abbreviations: hCoV, human coronavirus; LOESS, locally estimated scatterplot smoothing; RSV, respiratory syncytial virus; SARS-CoV-2, severe acute respiratory syndrome coronavirus 2.

## Supplementary Figure 3. Viral loads for participants with MA RSV LRTI (protocol defined) through Day 511 according to RSV coinfection status (A) and hospitalization status (B and C).


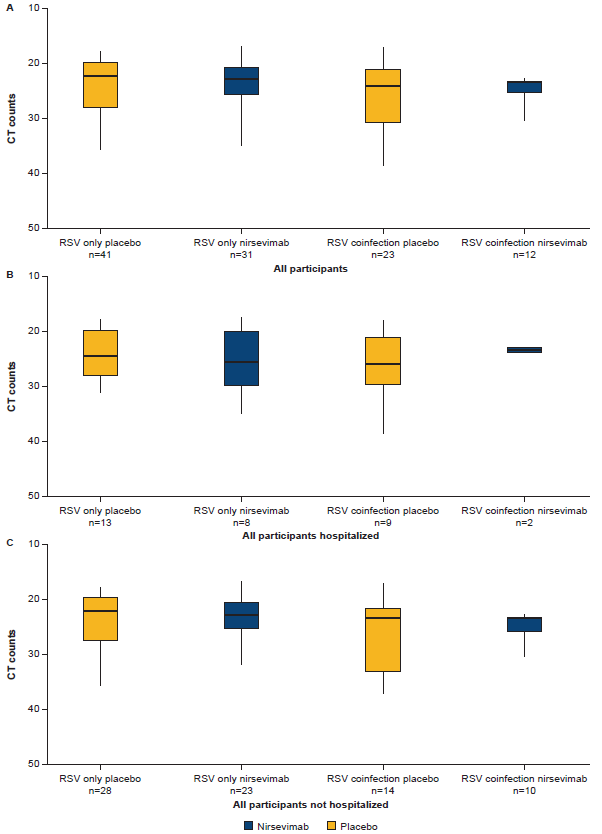


ITT Population, All participants. Boxes indicate the 25th and 75th percentiles, with the middle line indicating the median value, and the whiskers represent the range. Abbreviations: CT, cycle threshold; ITT, intention to treat; LTRI, lower respiratory tract infection; MA, medically attended; RSV, respiratory syncytial virus.

## Supplementary Figure 4. Severity of all MA LRTIs (any cause) through day 511 by treatment group according to hospitalization or no hospitalization for non-RSV respiratory viruses.


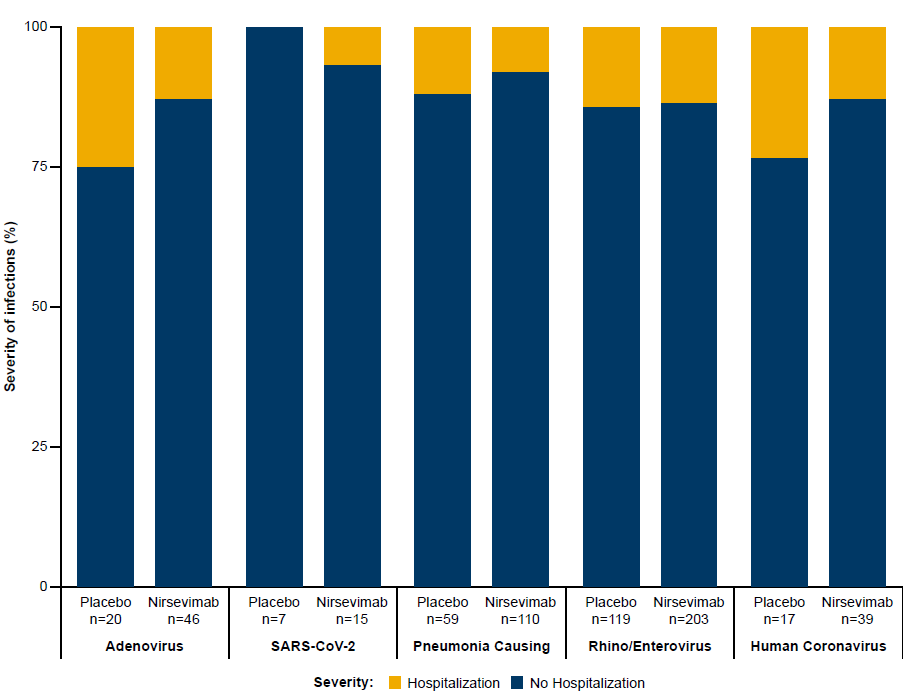


Hospitalized: Life-threatening (Requiring respiratory support by mechanical ventilation/CPAP/HFNC or PICU); Very severe (Hospitalized for RSV lower respiratory tract infection and requirement of oxygen or IV supply but not requiring mechanical ventilation/CPAP/HFNC or PICU); Severe (Hospitalized, but not requiring oxygen or IV or mechanical ventilation/CPAP/HFNC/PICU [intensive care]. Not hospitalized: Moderate (ReSViNET score >6, but not hospitalized); Mild (ReSViNET score ≤6, but not hospitalized).

Abbreviations: CPAP, continuous positive airway pressure; hCoV, human coronavirus; HFNC, High-flow nasal cannula; IV, intravenous; MA-LRTI, medically attended lower respiratory tract infection; PICU, pediatric intensive care unit; SARS-CoV-2, severe acute respiratory syndrome coronavirus 2.
